# Individual-Level Interventions for Decreasing Job-Related Stress and Enhancing Coping Strategies Among Nurses: A Systematic Review

**DOI:** 10.3389/fpsyg.2021.708696

**Published:** 2021-07-19

**Authors:** Maria Velana, Gerhard Rinkenauer

**Affiliations:** Department of Ergonomics, Leibniz Research Center for Working Environment and Human Factors (IfADo) at Dortmund University of Technology, Dortmund, Germany

**Keywords:** nurses, job-related stress, stress management, coping strategies, technology-delivered interventions, objective measurement tools

## Abstract

**Background:** Nurses are facing unprecedented amounts of pressure because of the ongoing global health challenges. Improving nurses' resilience to job-related stress and enhancing their strategies to cope effectively with stressors are key issues facing many health care institutions during the COVID-19 pandemic. This literature review aimed to: a) provide a thorough overview of individual-level interventions for stress management among nurses, b) identify measurement tools utilized to evaluate nurses' stress level, and c) provide the best evidence-based recommendations for future research and practice adapted to the current restrictions.

**Design:** Systematic review.

**Data Sources:** Studies published between January 2000 and October 2020 were retrieved from the following sources: EBSCOhost, Dortmund University Library, PubMed, Medline, Google Scholar, *Applied Nursing Research*, and reference lists from relevant articles.

**Review methods:** Individual-level interventions with a control group or a placebo intervention were included in the final sample. Primary outcome was defined as a change in individual stress level or stress symptoms which were measured by objective or subjective instruments with evidence of validity. Articles published in English or German were included in the present review.

**Results:** In total, 27 relevant studies were included into the current review. There are some indications that technology-delivered interventions with relaxation and stress management interventions comprising cognitive-behavioral components might be effective in decreasing stress among nurses and improving their well-being. Furthermore, although there were some attempts to collect objectively measured parameters for assessing the primary outcome of stress, the majority of the interventions utilized self-reported stress scales.

**Conclusion:** A wide range of interventions are available for nurses. However, it is of utmost importance to develop and implement stress management programs that are conveniently accessible in the workplace and above all, meet the current restrictions for minimizing human contacts. To this end, innovative interventions delivered through digital technology, such as virtual reality, seem to be a promising solution for combating the detrimental impact of stress on nurses. Special attention should be also paid to applying standardized objective measurement tools to allow the assessment of sensitive physiological indices and the generalizability of scientific knowledge.

## Introduction

Nowadays, nurses are facing unprecedented amounts of pressure not only due to the growing global health demands but also the current COVID-19 pandemic. The rapid spread of the virus during the last year has placed a huge burden on many health care systems scrambling to cover the needs for intensive care unit beds, personal protective equipment for both health care professionals and patients, and offer high-quality health services to their end-users (Arnetz et al., 2020). The current pandemic outbreak can be considered as a stressor that has significantly affected nurses' mental health. According to stress literature, when the existence of an organism is threatened by exposure to either a physiological or a psychological stressor, the system reacts to the stressful situation in a generalized, complex fashion (Matousek et al., 2010). The transactional model of stress postulates that one's perception of a stressor will depend on the degree to which an individual assesses this stressor as meaningful and relevant to them in a given context (Lazarus and Folkman, 1984). Further, meaningfulness will shape the strength of one's reaction to the event. On physiological level, stress triggers an initial activation of the sympathetic nervous and adrenomedullary systems resulting in increases in cardiac activity (Zhang et al., 2018). Furthermore, activation of the hypothalamus-pituitary-adrenal (HPA) axis provokes endocrine and immune changes leading to the release of cortisol and cytokines in an effort to re-establish body balance (Matousek et al., 2010). Stress research has showed that stressful events at the workplace can cause high physiological and psychological workload which can lead, in turn, to serious health problems and burnout [Lazarus and Folkman, 1984; Eatough et al., 2011; Velana and Rinkenauer (in press)].

In stressful occupations, such as nursing, the experience of stress deriving from lack of social support, heavy workload, conflicts with colleagues and other critical job-related factors is strongly linked with poor health (Tyler and Cushway, 1995). In fact, scientific evidence supports such an association, as stress in nurses can cause health problems and psychosomatic disorders, absenteeism, workplace injuries and errors related to patient care (Shirey, 2006). Moreover, study findings have indicated that nursing profession is associated with high rates of psychiatric outpatient referrals and suicide (Jones, 1987). More recently, in a cross-sectional study among 1257 Chinese health care providers from 34 national hospitals, female nurses, especially those who work in Wuhan, reported more severe symptoms on distress, anxiety and depression as compared to physicians (Lai et al., 2020). Another cross-sectional study that was conducted in Germany, indicated that nurses reported higher levels of stress and subjective burden as well as lower levels of job satisfaction and experienced support than physicians (Kramer et al., 2020). In a broader context, the present health crisis urges attention on nurses' mental health and on the strategies that should be developed to enhance their well-being and quality of life. Therefore, developing and implementing innovative approaches may be a best practice policy to reduce stress and improve health (Hatcher et al., 2006).

Improving resilience to stress and enhancing nurse's strategies to respond effectively to stressors are key issues facing many health care institutions during the COVID-19 pandemic. Resilience is considered one's ability to recover easily and quickly from adverse circumstances that happen over the course of their life (Zautra et al., 2010). Applying this notion to an organizational environment, resilience to stress implies that, in general, employees can respond in a productive way when encounter significant job-related changes or pressure to reach outcomes (Home and Orr, 1998). In nursing profession, this can be proved particularly challenging, as nurses often have to deal with human suffering, interpersonal difficulties and other job-related issues such as bullying and violence (Jackson et al., 2007), conditions that are associated with high levels of stress and demand adequate personal resources and coping strategies. Hence, enhancing nurses' resources and support may have the potential to develop their capacities to deal with stress and workplace adversities. The last years health care organizations around the globe have developed and implemented various individual-level interventions and strategies to empower employees to tackle setbacks at the workplace. In particular, interest has been growing in highlighting the effects of interventions on stress management and improving nurses' mental health, such as mindfulness, meditation and relaxation techniques (Delgado et al., 2017; Ghawadra et al., 2019). Furthermore, in his study, Cottrell (2001) showed that focused interventions for mental health nurses can enhance work-related factors, such as job satisfaction, and ameliorate stressors at workplace. Another literature review revealed that different stress management programs, such as training in therapeutic skills or in behavioral techniques, may help nurses address stress (Edwards and Burnard, 2003). Nevertheless, the rigor of a number of studies has induced methodological weaknesses related, for example, to measurement tools utilized, study sample size or statistical methods used for the analysis of their results. Although many interventions appear promising to effectively decrease stress and improve well-being among nurses, there is another body of evidence that indicates only moderate or no intervention effects, and calls for further research in this field (Chesak et al., 2019; Li et al., 2019). In light of the current contact restrictions, it still remains unclear what strategies would be suitable to tackle job-related stress throughout the COVID-19 crisis and the era after it.

Since nurses experience high levels of stress at the workplace, it is of vital importance to review and systematically evaluate the studies that utilized various individual-level strategies as a method to reduce their stress. Therefore, the present literature review aims to address the following issues: (a) to provide a thorough overview of the stress management interventions targeted at helping nurses develop skills to cope effectively with stress, (b) to identify measurement tools utilized to evaluate nurses' stress level (i.e., subjective and objectively measured parameters), and (c) to provide future research and practice with fruitful evidence-based directions adapted to the current restrictions.

## Methods

### Search Strategies

In an effort to examine the current state of the science regarding individual-level interventions for reducing job-related stress in nurses, studies published between January 2000 and October 2020 were retrieved from the following sources: EBSCOhost, Dortmund University Library, PubMed, Medline, Google Scholar, *Applied Nursing Research*, and reference lists from relevant articles. Although job-related stress in nurses and their coping strategies has drawn researchers' attention for over 30 years (e.g., Jones, 1987; Tyler and Cushway, 1995), the authors decided to focus on the advances of the last two decades so that they would provide the readers with a thorough insight about the topic and at the same time, suggest up-to-date directions for future studies that would align with the present global challenges. Specific search strategies were developed for each database to identify relevant interventions for this literature review. The present literature search was performed utilizing the following key terms: nurses, stress reduction interventions, stress management programs, (workplace) mental health interventions, job stress and coping strategies, in various combinations. Particularly, the following keyword combinations were applied: nurses AND stress reduction interventions, nurses AND stress management programs AND job stress, nurses AND mental health interventions AND job stress AND coping strategies, nurses AND workplace mental health interventions AND job stress. Special attention was paid to the differences among the databases in regard to vocabulary and syntax rules. The search was performed in November 2020.

The review protocol included two main steps: the first step involved reviewing of databases, while the second step consisted of identifying and screening all relevant papers according to inclusion and exclusion criteria. In order to ensure consistency and rigor, the guideline of “The Preferred Reporting Items for Systematic Reviews and Meta-Analyses (PRISMA)” was utilized (Moher et al., 2009).

### Inclusion Criteria

According to research literature, nurses are more likely to experience higher levels of job-related stress compared to other hospital employees and health professionals (Moustaka and Constantinidis, 2010; Golshiri et al., 2012; Kramer et al., 2020). Although stressful events and emergency situations seem to be common phenomena for hospital employees, nurses are prone to stress because of the psychological, physical and social attributes embedded in their occupational sector (Moustaka and Constantinidis, 2010). Building on this notion, only empirical research articles that focused on nurses who work in health care facilities, aiming at stress reduction were included in the final sample. Furthermore, two individual-level interventions should have been compared to each other, or an individual-level intervention should have been be compared to a control or placebo group in a prospective way. For instance, randomized controlled studies with or without random assignment, studies with quasi-experimental design and pre-posttest design studies with control group and/or placebo cohort were considered for further evaluation. Studies were also considered for further analysis, if the components of the stress management intervention, such as methods, frequency and duration of the intervention, were clearly described. Primary outcome was defined as a change in individual stress level or stress symptoms which were measured by objective or subjective instruments with evidence of validity. Secondary outcomes could be, but not limited to: burnout, depression, anxiety, quality of life, job satisfaction, etc. Articles published in English or German were included in the present review.

### Exclusion Criteria

Interventions that focused not only on nurses (e.g., nursing students, nurse aids) or studies that included other health care professionals were excluded from this review. Other exclusion criteria were pure qualitative studies, one group pre-post designs, studies aiming at organization level changes and articles published earlier than 2000.

### Data Extraction

Data were extracted and formatted based on the review aim utilizing a pre-defined data extraction worksheet in Excel. Table 1 summarizes all relevant characteristics of identified studies. Particularly, the headings include: (1) study characteristics (author, year of publication, place of study, sample size and setting), and (2) intervention characteristics (design, duration and components, measurement tools, follow-up, and main findings). This process was checked by the two review authors.

**Table 1:** Summary of articles included in the current literature review.

**Author (year) and place**	**Sample size and setting**	**Design**	**Duration and components**	**Measurement tools**	**Follow-up**	**Main findings**
Alkhawaldeh et al. (2020), Jordan	Total (*N* = 184): Treatment (*n* = 92); Control (*n* = 92); CHCC	Cluster-RCT	2-week SMIP (six 2-hour sessions twice a day) Waitlist control group	NSS Brief COPE Scale	Baseline, after the intervention and follow-up 2 months after the intervention	↓ stress after the intervention and at follow-up (*p* =0.001) ↑ coping strategies after intervention and at follow-up (*p* =0.001)
Bahmanzadeh and Haji Alizadeh (2017), Iran	Total (*N* = 30): Treatment (*n* = 15); Control (*n* = 15); Hospital	Quasi-experimental, pretest-posttest study with control	8-week cognitive-behavioral training (75 min/week) Passive control group	DASS WHOQOL-BREF	Baseline and after the intervention	↓ stress and anxiety (*p* < 0.05) ↑ quality of life (*p* < 0.05)
Bernburg et al. (2019), Germany	Total (*N* = 86): Treatment (*n* = 44); Control (*n* = 42); Psychiatric hospitals	Randomized controlled pilot study	12-week mental health program (1.5–2 h/week) Waitlist control group	PSQ ERSQ-27 BRCS SWOP-K9 QRI Evaluation form	Baseline and three follow-ups over a period of 36 weeks (after 3 months, T1; after 6 months, T2; after 12 months, T3)	↓ stress at T1 (*p* < 0.01), T2 (*p* < 0.01) and T3 (*p* < 0.01) ↑ for all additional outcomes at T1, T2and T3 (*p* < 0.05) ↑ program evaluation
Bernburg et al. (2020), Germany	Total (*N* = 94): Treatment (*n* = 47); Control (*n* = 47); Hospital	RCT	12-week work-related self-care skill training (1.5 h/week) Waitlist control group	PSQ COPSOQ MBI-EE ERSQ-27 Evaluation form	Baseline and three follow-ups over a period of 36 weeks (after 3 months, T1; after 6 months, T2; after 12 months, T3)	↓ stress at T1 (*p* < 0.001), T2 (*p* < 0.001) and T3 (*p* < 0.01) ↑ emotional exhaustion and emotion regulation skills at T1, T2and T3 (*p* < 0.05) ↑ job satisfaction at T1 (*p* = 0.01) ↑ program evaluation
Calder Calisi (2017), USA	Total (*N* = 46): Treatment (*n* =24); Control (*n* =22); General Hospital	Randomized, waitlist control design	8-week RR (45-min session; self-practice 10-20 min twice a day) Waitlist control group	STAI Semantic differential scales	Baseline and after the intervention	↓ anxiety (*p* = 0.02) and stress (*p* = 0.003) ↑ confidence in teaching RR (*p* < 0.001)
Cohen-Katz et al. (2005), USA	Total (*N* = 27): Treatment (*n* = 14); Control (*n* = 13); Academic-community—based Hospital	Pretest-posttest control group design with randomization	8-week MBSR program (2.5 h/week; home-based practice 6 days/week) Waitlist control group	MBI BSI MAAS Evaluation form	Baseline (T1), after the intervention (T2) and 3-month follow-up (T3)	↓ emotional exhaustion (MBI) at T2 and T3 (*p* < 0.05) ↑ MAAS at T2 (*p* = 0.004) and T3 (*p* = 0.002) ↑ program evaluation
Collier et al. (2018), USA and UK	Total (*N* = 16): Treatment (*n* = 8); Control (*n* = 8); Psychiatric inpatient unit (Hospital and Mental Health Services)	Randomized trial	4-week MSET (two 40-min sessions/week) Control group; standard unit lounge	Pulse rate STAI POMS Evaluation form	Before and after each session	↓ pulse rate (*p* = 0.001), in State scale (*p* < 0.001) and Trait scale (*p* = 0.015) ↑ Confusion Bewilderment sub-scale of POMS (*p* = 0.004) ↑ program evaluation
Ghawadra et al. (2020), Malaysia	Total (*N* = 249: Treatment (*n* = 123); Control (*n* = 126); Teaching hospital	RCT	4-week mindfulness-based intervention (2-hour workshop; self-practice guided by a website) Waitlist control group	DASS-21 JSS MAAS	Baseline, after the intervention and follow-up 8 weeks after the intervention	↓ stress (*p* < 0.001), anxiety (p = 0.001) and depression (*p* < 0.001) over time ↑ mindfulness (*p* < 0.001) over time ↑ job satisfaction (*p* < 0.001)
Gollwitzer et al. (2018), Germany	Total (*N* = 129): Treatment (MCII, *n* = 41; IIMCII, *n* = 41); Control (*n* = 47); Health institutions	Randomized factorial design	3-week MCII (mental exercise daily) 3-week IIMCII (modified mental exercise daily) Passive control group	PSQ-20 Physical symptoms subscale of BOSS II UWES-9	Baseline and after the intervention	↓ stress in the MCII group compared to the control group (*p* = 0.019) ↑ work engagement in the MCII group as compared to the IIMCII (*p* = 0.029) and the control group (*p* = 0.046)
Hersch et al. (2016), USA	Total (*N* = 104): Treatment (*n* = 52); Control (*n* = 52); Hospital	RCT	12-week web-based *BREATHE* program (unlimited online access) Waitlist control group	NSS Symptoms of Distress Coping with Stress WLQ Use of Substances for Stress Relief Drinking Quantity and Frequency Understanding Depression and Anxiety Nurses Job Satisfaction Scale	Baseline and after the intervention	↓ stress (*p* < 0.001) and NSS sub-scales (*p* < 0.05) apart from sub-scale lack of support No effect on additional outcomes
Hsieh et al. (2020), Taiwan	Total (*N* = 135): Treatment (BT, *n* = 49; SDBT, *n* = 47) Control (*n* = 39); Psychiatric wards	Quasi-experimental study	6-week BT (1 h/week) 6-week SDBT (once a week) Waitlist control group	CES-D OSI-2 RS Physiological parameters (HRV: SDNN, LF, HF; RR) Rehabilitation strength chart Simplified health scale	Baseline and after the intervention	↓ stress (*p* =0.013) in SDBT group ↑ depressive symptoms (*p* < 0.001), resilience (*p* < 0.001), and respiration rate for BT (*p* < 0.001) and SDBT (*p* = 0.002)
Hwang and Jo (2019), Korea	Total (*N* = 60): Treatment (*n* = 30); Control (*n* = 30); College hospital	RCT	4-week app-based stress-management program (twice a week for at least 10 min) Waitlist control group	PSSKOSS(PHQ)-9(GAD)-7Korean-Emotional Labor scaleWHO-5 Well-Being IndexSelf-efficacy (Likert) scaleEvaluation form	Baseline and after the intervention	↓ stress (PSS, *p* = 0.035; KOSS, *p* = 0.04) and emotional labor (*p* = 0.027)↑ well-being (*p* = 0.005) and self-efficacy (*p* = 0.025)↑ program evaluation
Kurebayashi et al. (2012), Brazil	Total (*N* = 75): Treatment (Needle group, *n* = 27; Seed group, *n* = 26); Control (*n* = 22); Teaching hospital	RCT	8-week auriculotherapy with needles (eight sessions, 5-10 min/week) 8-week auriculotherapy with seeds (eight sessions, 5-10 min/week) Passive control group	LSS Folkman and Lazarus' Ways of Coping questionnaire	Baseline, after 4 sessions, after 8 sessions and follow-up 15 days after the intervention	↓ stress after 8 sessions (*p* = 0.020) and at follow-up (p = 0.003) in the needle group ↑ Distancing domain (*p* = 0.039) and Confrontive Coping domain (*p* = 0.029) at follow-up in the needle group ↑ Seeking Social Support domain (*p* = 0.022) after 8 sessions in the seed group
Lary et al. (2019), Iran	Total (*N* = 70): Treatment (*n* = 35); Control (*n* = 35); Teaching hospital	Quasi-experimental study	6-week McNamara educational method (1 h/week) Waitlist Control group	SRI	Baseline, after the intervention and follow-up 8 weeks after the intervention	↓ stress (*p* = 0.021) over time
Lin et al. (2019), China	Total (*N* = 90): Treatment (*n* = 44); Control (*n* = 46); General hospital	Randomized controlled design	Modified 8-week MBSR program (group sessions 2 h/week and home-based practice 20 min × 6 days/week) Waitlist control group	PSS PANAS CD-RISC MMSS	Baseline, after the intervention (T1), and follow-up 3 months later (T2)	↓ stress and negative affect at T1 (*p* < 0.01) and T2 (*p* < 0.05) respectively ↑ positive affect at T1 and T2 (*p* < 0.05) and resilience at T2 (*p* < 0.05) No effect on job satisfaction
McElligott et al. (2003), USA	Total (*N* = 20): Treatment (*n* = 12); Control (*n* = 8); University Hospital	Quasi-experimental design	4-week AMMA therapy (1 h/week) Control group; 4-week STTP	Physiologic Parameters (blood pressure, heart rate, pulse oximetry, and skin temperature) VAS Evaluation interview questionnaires	Baseline, before and after each treatment, and at completion of the study	↓ anxiety over time No effect on physiologic parameters ↑ program evaluation
Moeini et al. (2011), Iran	Total (*N* = 58): Treatment (*n* = 29); Control (*n* = 29); Training hospital	Quasi-experimental study	3-week cognitive-behavioral program based on PRECEDE model (five 60-90 min sessions) Passive control group	NSS Questionnaire based on PRECEDE model Evaluation form	Baseline and follow-up 1.5 months after the intervention	↓ stress (*p* < 0.001) ↑ PRECEDE model constructs and stress management behaviors (*p* < 0.001)
Nazari et al. (2015), Iran	Total (*N* = 66): Treatment (n = 33); Control (*n* = 33); ICUs (Hospital)	RCT	4-week massage therapy (25-min sessions twice a week) Passive control group	OSI	Baseline, after the intervention and follow-up 2 weeks after the intervention	↓ stress (*p* < 0.001) and subscale scores (*p* < 0.05) over time
Niva et al. (2020), India	Total (*N* = 30): Treatment (*n* = 15); Control (*n* = 15); Tertiary care hospital	RCT	Mahamantra chanting intervention for 45 days (20 min/day) Passive control group	Stress biomarkers (Serum cortisol, DHEA-S, SAA) Biochemical parameters (Glucose and lipid profile)	Baseline and follow-up after 2 menstrual cycles after the intervention	↓ serum cortisol (*p* = 0.012), SAA level (*p* = 0.04), glucose (*p* = 0.001), HbA1c (*p* = 0.041), total cholesterol (*p* < 0.001), LDLc (*p* < 0.001) and TGL (*p* = 0.17) ↑ HDLc (*p* = 0.033)
Orly et al. (2012), Israel	Total (*N* = 36): Treatment (*n* = 20); Control (*n* = 16); Hospital	Pre-posttest design study with control	16-week CBI course (4 h/week) and five job-related 3-hour seminars Control group; five job-related 3-hour seminars	SOC PSS POMS	Baseline and after the intervention	↓ stress (*p* < 0.05) and POMS fatigue (*p* < 0.05) ↑ SOC (*p* < 0.05) and POMS vigor (*p* < 0.05)
Palumbo et al. (2012), USA	Total (*N* = 14): Treatment (*n* = 7); Control (*n* = 7); Hospital	RCT	15-week Tai Chi program (group practice 45 min/week and self-practice at least 10 min × 4 days /week) Passive control group	SF-36 Health Survey NSS PSS Sit-and-reach test Isometric knee extensor strength test dynamometer Functional reach test Nordic Musculoskeletal Questionnaire WLQ Work absenteeism	Baseline and after the intervention	No effect on stress ↑ work productivity (*p* = 0.03) and functional reach (*p* < 0.01)
Prado et al. (2018), Brazil	Total (*N* = 168): Treatment (Auriculotherapy, *n* = 56; Placebo, *n* = 56); Control (*n* = 56); Hospital	Randomized, single-blind, controlled trial	Auriculotherapy with stress points (12 sessions, twice a week) Sham auriculotherapy with sham points (12 sessions, twice a week) Waitlist control group	LSS	Baseline, after eight sessions, 12 sessions and follow-up 15 days after the end of the applications	↓ stress in the treatment group after eight sessions and at follow-up (*p* < 0.001) ↓stress in the placebo group after 12 sessions (*p* < 0.001) and at follow-up (*p* < 0.05)
Singh and Jain (2017), India	Total (*N* = 40): Treatment (*n* = 20); Control (*n* = 20); Hospital	Pre-posttest design with control	Self-help intervention (four 30-min sessions with an interval of 10 days) Passive control group	Psychosocial Stress Questionnaire Occupational Stress Index	Baseline and after the intervention	↓ occupational stress and in psychosocial stress (*p* < 0.01)
Villani et al. (2013), Italy	Total (*N* = 30): Treatment (*n* = 15); Control (*n* = 15); Oncology hospital	Between-subjects design	4-week M-SIT (15-min sessions twice a week) Control group; neutral stimuli (15-min sessions twice a week)	MSP STAI COPE-4 JCQ	Baseline, before and after each session, after the intervention	↑ state anxiety (*p* < 0.001), trait anxiety (*p* = 0.041) and coping skills acquisition (*p* < 0.05)
Walker (2006), USA	Total (*N* = 98): Treatment (*n* = 58); Control (*n* = 40); Hospital	Quasi-experimental design	4-week HRTT HeartTouch technique (3-hour educational session; self-practice; 1-hour session 2 weeks after the initial session; final follow-up session) Control group (2-hour educational session; final follow-up session)	PSS SWB DRS Diary HeartTouch questionnaire	Baseline and after the intervention	↓ stress (*p* < 0.001), and ↑ hardiness (*p* < 0.001) and spiritual well-being (*p* < 0.05) in the treatment group ↓ stress (*p* < 0.001) and ↑ hardiness (*p* < 0.05) in the control group
Wang et al. (2017), Taiwan	Total (*N* = 78): Treatment (MBSR, *n* = 35; Humanities class, *n* = 35); Control (*n* = 12); Hospital	Quasi-experimental design	8-week MBSR intervention (3 h/week) 8-week humanities class (3 h/week) Passive control group	FFMQ NSC	Baseline (T0), after 1st month of MBSR (T1), after the intervention (T2), at 3rd month (T3) and 6th month (T4)	↑ mindfulness (*p* = 0.031) in the MBSR group
Yang et al. (2018), China	Total (*N* = 100): Treatment (*n* = 50); Control (*n* = 50); Psychiatric departments	Pre-posttest design with control	8-week MBSR therapy (once a week; either group training or home-based practice) Control group; routine psychological support	SCL-90 SDS SAS NSS	Baseline and after the intervention	↓ stress, anxiety and depression scores (*p* < 0.001) ↑ mental health (*p* < 0.001)

*↓ decrease, ↑ improvement or positive. AMMA therapy, a healing art of therapeutic massage; BOSS II, Burnout Screening Scales II inventory; Brief COPE Scale, Brief Coping Orientations for Experienced Problems Scale; BRCS, Brief Resilient Coping Scale; BREATHE, Stress Management for Nurses program; BSI, Brief Symptom Inventory; BT, biofeedback training; CBI, cognitive-behavioral intervention; CD-RISC, Connor–Davidson Resilience Scale; CES-D, Center for Epidemiological Studies Depression; CHCC, Comprehensive Health Care Centers; COPE, Brief Coping Orientation to Problems Experienced questionnaire; COPSOQ, Copenhagen Psychosocial Questionnaire; DASS, Depression, Anxiety and Stress Scale; DHEA-S, sulphated metabolite of dehydroxyepiandrosterone; DRS, Dispositional Resilience Scale; ERSQ-27, Emotion Regulation Skills Questionnaire; FFMQ, five facet mindfulness questionnaire; GAD-7, Generalized Anxiety Disorder; HbA1c, glycated hemoglobin; HDLc, high density lipoprotein cholesterol; HF, high frequency; HRTT, HeartTouch technique; HRV, heart rate variability; IIMCII, modified strategy of MCII; JCQ, Job Content Questionnaire; JSS, Job Satisfaction Scale for Nurses; KOSS, Korean Occupational Stress Scale; LDLc, low density lipoprotein cholesterol; LF, low frequency; LSS, Stress Symptom List; MAAS, Mindfulness Attention Awareness Scale; MBI-EE, emotional exhaustion subscale of the Maslach Burnout Inventory; MBSR, mindfulness-based stress reduction; MCII, mental contrasting with implementation intentions; MMSS, McCloskey/Mueller satisfaction scale; MSET, multisensory environmental therapy; M-SIT, Mobile Stress Inoculation Training; MSP, Mesure du Stress Psychologique; NSC, nurse stress checklist; NSS, Nursing Stress Scale; OSI, Occupational Stress Inventory; OSI-2, Occupational Stress Indicator; PANAS, positive and negative affect schedule; PHNs, public health nurses; PHQ-9, Patient Health Questionnaire; POMS, Profile of Mood States; PRECEDE, predisposing, reinforcing and enabling factors; PSQ, Perceived Stress Questionnaire; PSQ-20, Perceived Stress Questionnaire-20; PSS, Perceived Stress Scale; QRI, German Quality of Relationship Inventory; RCT, Randomized controlled trial; RR, Relaxation Response; RR, respiration rate; RS, Resilience Scale; SAA, salivary alpha amylase; SAD, stress, anxiety or depression; SAS, Self-rating anxiety scale; SCL-90, Symptom Checklist-90; SDBT, smartphone-delivered biofeedback training; SDNN, standard deviation of normal to normal; SDS, Self-rating depression scale; SMIP, stress management interventional program; SOC, Sense of Coherence; SRI, Stress Response Inventory; STAI, State Trait Anxiety Inventory; STTP, Standardized Touch Therapy Protocol; SWB, Spiritual Well-Being Scale; SWOP-K9, Self-Efficacy, Optimism and Pessimism questionnaire; TGL, triglycerides; UWES-9, Utrecht Work Engagement Scale; VAS, Visual Analog Scale; WHOQOL-BREF, World Health Organization Quality-of-Life Scale; WLQ, Work Limitations Questionnaire*.

## Results

### Search Outcome

Based on inclusion and exclusion criteria, first the two review authors independently screened the titles and abstracts of all relevant articles. Next, full-text versions of all potentially eligible articles were evaluated independently by the two authors to define whether all inclusion criteria were met. External experts would be consulted to achieve a consensus in case of disagreement. However, this was not the case for the current review.

**Figure 1:**
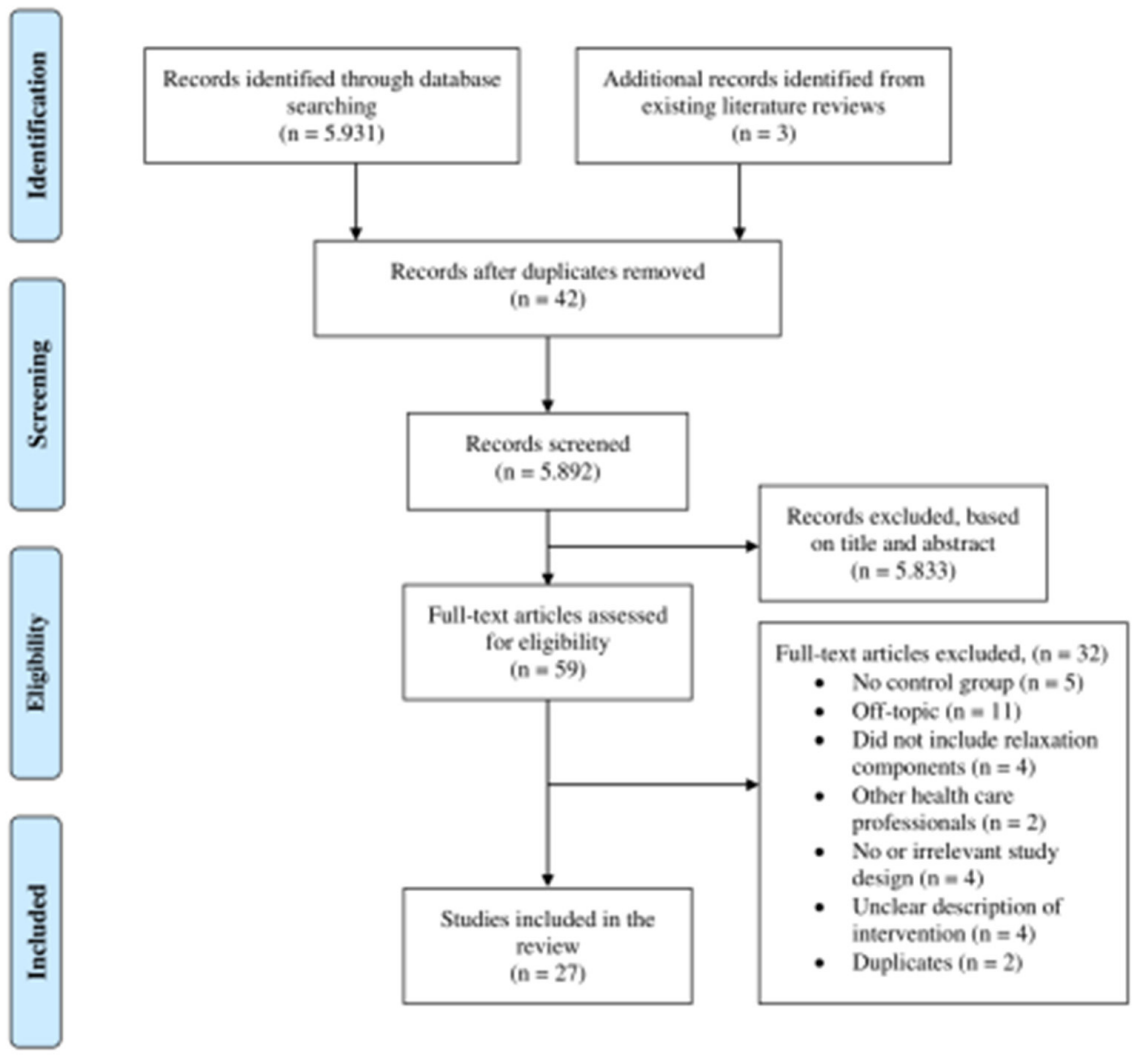
Search strategy for the inclusion and exclusion of articles based on a modified version of PRISMA flow diagram (Moher et al., 2009).

In total, records 5,931 were retrieved. Additionally, three studies were identified from the reference lists of previously published literature reviews (Chesak et al., 2019; Ghawadra et al., 2019; Bakker et al., 2020). 5,892 records were left for screening after removing duplicates. Next, titles and abstracts were assessed and 5,833 were excluded, leaving 59 potentially relevant articles. Screening of the full-text articles indicated that 32 did not fulfill the inclusion criteria, leaving 27 studies for this literature review. Figure 1 tracks the selection process of the relevant studies utilizing a modified version of the PRISMA flow diagram (Moher et al., 2009).

## Study Characteristics

### Place of Study

Four of these studies were carried out in European countries, and the other studies were from different countries: Brazil (*n* = 2), China (*n* = 2), India (*n* = 2), Iran (*n* = 4), Israel (*n* = 1), Jordan (*n* = 1), Korea (*n* = 1), Malaysia (*n* = 1), Taiwan (*n* = 2), USA (*n* = 6). Furthermore, one study took place in health care institutions based in two different countries, namely the USA and the UK.

### Sample Size and Setting

The sample size of the included studies varied widely, from 14 participants (Palumbo et al., 2012) to 249 participants (Ghawadra et al., 2020). Participant work settings included hospitals (McElligott et al., 2003; Cohen-Katz et al., 2005; Walker, 2006; Moeini et al., 2011; Kurebayashi et al., 2012; Orly et al., 2012; Palumbo et al., 2012; Villani et al., 2013; Hersch et al., 2016; Bahmanzadeh and Haji Alizadeh, 2017; Calder Calisi, 2017; Singh and Jain, 2017; Wang et al., 2017; Prado et al., 2018; Hwang and Jo, 2019; Lary et al., 2019; Lin et al., 2019; Bernburg et al., 2020; Ghawadra et al., 2020; Niva et al., 2020), settings that focus on mental health (Collier et al., 2018; Yang et al., 2018; Bernburg et al., 2019; Hsieh et al., 2020), intensive care units (Nazari et al., 2015), and health care institutions with different organizational and hierarchical structures (Gollwitzer et al., 2018; Alkhawaldeh et al., 2020).

## Intervention Characteristics

### Design

Fifteen studies used a randomized controlled trial to study the effects of the treatment on nurses' stress level (Kurebayashi et al., 2012; Palumbo et al., 2012; Nazari et al., 2015; Hersch et al., 2016; Calder Calisi, 2017; Collier et al., 2018; Gollwitzer et al., 2018; Prado et al., 2018; Bernburg et al., 2019, 2020; Hwang and Jo, 2019; Lin et al., 2019; Alkhawaldeh et al., 2020; Ghawadra et al., 2020; Niva et al., 2020). However, only three studies employed blind procedures (Prado et al., 2018; Alkhawaldeh et al., 2020; Bernburg et al., 2020). The rest of these studies either did not use blinding procedures or did not report any attempts of blinding. Seven studies used a quasi-experimental design (McElligott et al., 2003; Walker, 2006; Moeini et al., 2011; Bahmanzadeh and Haji Alizadeh, 2017; Wang et al., 2017; Lary et al., 2019; Hsieh et al., 2020) and the remaining five studies utilized a pre-posttest design with control group (Cohen-Katz et al., 2005; Orly et al., 2012; Villani et al., 2013; Singh and Jain, 2017; Yang et al., 2018). Out of the 27 studies included in the review, only eight studies conducted a psychological screening to define whether participants suffered from moderate or high levels of psychological stress prior to being invited to take part in the research study (Kurebayashi et al., 2012; Villani et al., 2013; Nazari et al., 2015; Singh and Jain, 2017; Prado et al., 2018; Yang et al., 2018; Ghawadra et al., 2020; Niva et al., 2020).

### Intervention Duration and Components

All the interventions included in the current review aimed at treatment of the individual. The intervention duration ranged from 2 weeks (Alkhawaldeh et al., 2020) to 16 weeks (Orly et al., 2012). As for the modalities, the most common interventions were technology-delivered interventions for stress management and mental health. Particularly, three studies implemented self-help programs guided by a website (Hersch et al., 2016; Gollwitzer et al., 2018; Ghawadra et al., 2020) and three additional interventions investigated the effectiveness of mobile phone-delivered programs for stress management (Villani et al., 2013; Hwang and Jo, 2019; Hsieh et al., 2020). Other commonly used modality types included mindfulness-based programs (Cohen-Katz et al., 2005; Walker, 2006; Wang et al., 2017; Yang et al., 2018; Lin et al., 2019), cognitive-behavioral interventions (Moeini et al., 2011; Orly et al., 2012; Bahmanzadeh and Haji Alizadeh, 2017), self-care interventions (Singh and Jain, 2017; Lary et al., 2019; Alkhawaldeh et al., 2020), auriculotherapy (Kurebayashi et al., 2012; Prado et al., 2018), massage (McElligott et al., 2003; Nazari et al., 2015), psychological competence trainings combined with cognitive-behavioral components (Bernburg et al., 2019, 2020), breathing exercises (Calder Calisi, 2017), chanting mantras (Niva et al., 2020), physical activity training (Palumbo et al., 2012) and multisensory environmental therapy (Collier et al., 2018).

### Measurement Tools and Follow-Up

Diverse instruments were used depending on the research aims of each study. The present review focuses on the subjective and objective assessment tools for measuring stress in nurses. Among the 27 articles, 18 different instruments were utilized to assess subjective stress experience. The most commonly used tools to record stress were the Perceived Stress Scale (PSS) and the Nursing Stress Scale (NSS), which were used in 10 studies. Other instruments that were frequently utilized, were the Perceived Stress Questionnaire (PSQ), the Stress Symptom List (LSS), the State Trait Anxiety Inventory (STAI), and the Depression, Anxiety, and Stress Scale (DASS). Furthermore, the primary outcome of stress was also objectively measured in four interventions. In particular, physiological parameters, such as blood pressure, pulse oximetry, skin temperature, respiration rate and cardiac response, were assessed to quantify stress level (McElligott et al., 2003; Collier et al., 2018; Hsieh et al., 2020). One study evaluated serum stress markers and metabolic parameters without utilizing self-report (Niva et al., 2020).

In these studies, assessments of the primary outcome were performed for all participants before the beginning of the intervention, at baseline. All the interventions repeated the stress assessment after the completion of the treatment. However, only in 11 interventions, the measurements were conducted for a longer period of time, following the participants after the end of the treatment (Cohen-Katz et al., 2005; Kurebayashi et al., 2012; Nazari et al., 2015; Wang et al., 2017; Prado et al., 2018; Bernburg et al., 2019, 2020; Lary et al., 2019; Lin et al., 2019; Alkhawaldeh et al., 2020; Ghawadra et al., 2020). The end point for these follow-up examinations ranged from 2 weeks (Kurebayashi et al., 2012; Nazari et al., 2015; Prado et al., 2018) to 36 weeks (Bernburg et al., 2019, 2020) after the intervention.

### Main Findings

Twenty-three studies showed that stress level decreased after the intervention. However, two studies measured perceived stress only either with Visual Analog Scale [VAS; (McElligott et al., 2003)] or State Trait Anxiety Inventory [STAI; (Collier et al., 2018)]. Although one intervention mentioned decrease in self-reported measures, they did not report *p* values (McElligott et al., 2003). Furthermore, four studies did not indicate changes in perceived stress (Cohen-Katz et al., 2005; Palumbo et al., 2012; Villani et al., 2013; Wang et al., 2017). On physiological level, it was shown that the treatment had a significant effect on objectively measured stress indices (Collier et al., 2018; Hsieh et al., 2020; Niva et al., 2020). Nevertheless, one study reported no changes in the measured physiological parameters (McElligott et al., 2003). Overall, nine studies indicated long-term decrease in perceived work-related stress (Kurebayashi et al., 2012; Nazari et al., 2015; Prado et al., 2018; Bernburg et al., 2019, 2020; Lary et al., 2019; Lin et al., 2019; Alkhawaldeh et al., 2020; Ghawadra et al., 2020).

## Discussion

This systematic review of the literature investigating individual-level interventions for stress management in nurses revealed a wide variety of programs that can be mainly classified into: (a) technology-based interventions for stress management and mental health either guided by a website or delivered through mobile phones, (b) mindfulness-based and spiritual interventions, (c) programs with cognitive-behavioral components, and (d) programs addressing body. In particular, there are some indications that technology-delivered interventions with relaxation components and stress management interventions comprising self-care skills, cognitive-behavioral components and relaxation might be effective in reducing stress among nurses and improving their mental well-being. In this direction, earlier research reviews have indicated that a wide range of interventions are available and seem promising for decreasing psychological distress (Delgado et al., 2017; Ghawadra et al., 2019; Bakker et al., 2020). Although evidence supports the effectiveness of these mechanisms in tackling stress, the rapid changes in health care systems and the unpreceded pressure that nurses experience, highlight the need to develop interventions adapted to the new overwhelming demands. Indeed, the prevalence of mental health problems among health care workers, especially during and after outbreaks, is high (Ricci-Cabello et al., 2020). Furthermore, these problems are usually associated with long-term mental health burden and thus, hinder the immediate response to health threats, such as the present COVID-19 crisis.

It is of utmost importance to develop and implement stress management interventions that are not only conveniently accessible in the workplace but also, they meet the strict conditions for minimizing human contacts. To this end, evidence-based interventions and self-care practices for those in immediate need delivered through digital technology seem to be a promising solution for combating the detrimental psychological and physiological impact among nurses. For example, there is recently an emerging body of studies that examines the implementation of a self-help virtual reality (VR) protocol to overcome the negative consequences of the quarantine by reliving stress among individuals (Riva and Riva, 2020; Riva et al., 2020). The protocol is designed to simulate a natural environment, while the user can perform daily exercises aiming at self-concertation and relaxation. By transferring this idea to a demanding workplace, where restrictions for social contact apply, it might be an effective way to enhance nurses' resilience and generally, improve their mental health. Therefore, future research will be needed to examine, if the continuous use of technology-based stress management and the refinement of its technological capabilities would lead to individually tailored self-help programs and to a more positive effect.

Furthermore, the most commonly utilized instrument for assessing the primary outcome of stress was self-reported scales. However, the current review of the literature identified some efforts from these interventions to include objectively measured parameters in order to explore the effect of strategies developed on physiological level. The experience of high job stress can cause alternations in the physiological processes that the human body mobilizes in an attempt to re-establish inner balance. In psychophysiological studies, the exposure to a stressful event is associated with intense cardiac activity (Johnston et al., 2016) which may be considered as a predisposing factor for the onset of lifestyle diseases, such as depression and heart disease. Additionally, the prolonged activation of the HPA axis can lead to increased concentrations of stress hormones harming the immune system (Aguilera, 2011; Herman et al., 2016).

The assessment of specific stress biomarkers could provide considerable advantages to future studies: such measures will allow researchers to define the outcomes of their interventions in a more systematic and precise way, taking into consideration the individual differences. It is well-known that people respond to potential stressors with great variability (McEwen, 1998). In this regard, diverse individual-related factors, such as gender, age, health status and personality characteristics, may regulate not only physiological reactivity to stress but also individuals' ability to activate their resources and cope with challenges. By capturing physiological responses, it would be possible to reveal which psychophysiological mechanisms are involved into resilience processes and how individual characteristics are interwoven in physiological traits. This may contribute, in turn, to a better understanding of human body and the implementation of effective stress management strategies based on objective indices. Moreover, concrete physiological outcomes can be directly associated with stress scales that may have implications for individual's health status, and can reduce confounding effects by response bias inherent in self-report ratings (Schnall et al., 1992; Bosma et al., 1997; McEwen, 1998). Therefore, future studies could utilize physiological parameters as indices for assessing their effectiveness to reduce stress and overcoming certain methodological issues of self-report.

There are some other issues that have been identified by the current literature review. The majority of the studies included have been designed and conducted in US, where the surrounding conditions and needs for mental health care among nurses may slightly differ from those exist around the globe. These scientific data and knowledge deriving from research on preventive programs may be usefully applied to the case of nurses in countries other than the US. However, the fact that different countries operate very different health care systems may imply that there are limitations in generalizing and integrating the study findings (Edwards and Burnard, 2003). In line with this, most authors of the identified interventions recognized the limited generalizability of their results. In fact, it was found that most of the interventions were developed for a clinical environment and in most cases, they reported a small sample size or a homogeneous study population. Nevertheless, no interventions were identified recruiting large random samples, for example, in nursing homes, elderly care or health care facilities for homeless, where nurses may have increased needs for help and be at higher risk for chronic stress and stress-related diseases. In general, research results should be generalized with caution and future studies may adjust their methods to the local conditions of health care professionals. Another issue related to generalizability of the study findings extracted is that, in many articles, p values were reported without including the effect size, which is a standardized measure to evaluate interventions' clinical utility. Therefore, this fact rendered the comparison of the results difficult and based on this one might question their generalizability to other settings. Although previous research on stress reduction in health care providers has also identified the same methodological problem (e.g., Edwards and Burnard, 2003; Bakker et al., 2020), it still concerns. Furthermore, future research may pay more attention into different nursing specialties by developing strategies to meet the demands of non-hospital-based institutions (Bakker et al., 2020). Another major limitation of the included studies was lack of long-term follow-up data. Although the majority of the studies effectively decreased work-related stress immediately after the intervention and highlighted the benefits of such interventions for enhancing nurse's mental health, only nine programs out of the 27 identified interventions indicated a long-term change in the measured outcome. Nurses might have shown temporary improvements in stress immediately following a stress management program but might have returned later to baseline levels, especially without continued support. Hence, future interventions are needed to include longer follow-up intervals that can more reliably indicate the extent of their effect.

### Strengths and Limitations of the Systematic Review

The present literature review provides the reader a thorough overview of the existing programs aimed at reducing stress in nurses and helping them develop adequate coping resources. The benefits of these interventions examined encourage the development of clinical applications and individual-level programs toward the particular group and outcome being measured. However, there is a number of limitations of the current review that should be considered. The diversity of the interventions and their treatment characteristics hindered a comparison of the different study findings, data-pooling and meta-analysis. This limitation might be overcome by the use of a tool for the quality assessment of the extracted studies. Future authors are highly encouraged to use such appraisal instruments. For the purpose of this review, only stress programs for nurses were identified and considered for further analysis. However, other groups of health care providers, such as nurse aids, novice nurses and nursing students, suffer from significant levels of distress and mental health problems (Mackenzie et al., 2006; Pulido-Martos et al., 2012; e.g., Chatterjee et al., 2014; Rathnayake and Ekanayaka, 2016). A deeper understanding of the needs of each health professional group may explain whether and why they respond to preventive programs differently. By addressing this important issue, it may help not only to implement individually tailored interventions, but also to create a basis to allocate effectively resources for interventions that alleviate stress in health care providers at all levels. Moreover, another limitation was that literature search was restricted to articles published in English or German, which might have caused the exclusion of relevant studies. In an attempt to limit the articles identified only to those that focused on interventions for nurses, authors used the term “nurses” for conducting their literature search and as a result, they might unknowingly exclude other relevant studies. This is a limitation that should be considered.

## Conclusion

Individual-level interventions and self-care strategies are core values for addressing the growing problem of stress among nurses. However, the question of what stress management programs would be effective to enhance nurses' personal resources to decrease stress throughout the pandemic still persists. This systematic review of the literature highlights the immediate need for evidence-based preventive interventions that may be delivered through digital technology combined with relaxation and cognitive-behavioral components to reduce stress and meet the current conditions that allow fewer human contacts. The integration of VR as a tool of stress management into mental health research has the potential to offer a radical transformation of the traditional intervention programs, allowing their users to meet the current restrictions for human contact. Therefore, special attention should be paid to advancing technology-based interventions that develop innovative self-help strategies, and to applying standardized objective measurement tools to allow the quantification of sensitive physiological indices and transferability of scientific knowledge. Further research is needed to develop preventive programs with long-term follow-up for those nurses who work in specialized care at non-hospital institutions so as to understand and meet their needs for mental health care. Nurses play an integral role in each health care system and should be provided with all these appropriate self-help strategies to enhance their resilience and create a healing environment, where they can better exhibit their skills and commitment to patients. Having healthy staff is essential to delivering high-quality health care and preventing serious mental health disorders at the workplace.

## Data Availability Statement

The original contributions presented in the study are included in the article, further inquiries can be directed to the corresponding author.

## Author Contributions

MV designed the study. MV, and GR performed the literature review, data extraction, and classification according to the pre-defined criteria. MV synthesized the study findings and drafted the manuscript. GR participated into the review process. Both authors contributed to the article, read and approved the submitted version.

## Conflict of Interest

The authors declare that the research was conducted in the absence of any commercial or financial relationships that could be construed as a potential conflict of interest.
